# Stent-graft surface movement after endovascular aneurysm repair: baseline parameters for prediction, and association with migration and stent-graft-related endoleaks

**DOI:** 10.1007/s00330-019-06282-w

**Published:** 2019-06-27

**Authors:** Ulrika Asenbaum, Maria Schoder, Ernst Schwartz, Georg Langs, Pascal Baltzer, Florian Wolf, Alexander M. Prusa, Christian Loewe, Richard Nolz

**Affiliations:** 1 Division of Cardiovascular and Interventional Radiology, Department of Bio-medical Imaging and Image-Guided Therapy, Medical University of Vienna - Vienna General Hospital, Waehringer Guertel 18-20, A-1090 Vienna, Austria; 2 Computational and Imaging Research Laboratory, Department of Bio-medical Imaging and Image-Guided Therapy, Medical University of Vienna, Vienna, Austria; 3 Department of Surgery, Medical University of Vienna, Vienna, Austria

**Keywords:** Aortic aneurysm, Abdominal aorta, Stent–graft, Computed tomography angiography, Movement risk factors

## Abstract

**Objectives:**

To evaluate the influence of baseline parameters on the occurrence of stent-graft surface movement after endovascular aneurysm repair (EVAR) and to investigate its association with migration and stent-graft-related endoleaks (srEL).

**Methods:**

In this retrospective, cross-sectional study, three-dimensional surface models of the stent-graft, delimited by landmarks using custom-built software, were derived from the pre-discharge and last follow-up computed tomography angiography (CTA). Stent-graft surface movement in the proximal anchoring zone between these examinations was considered significant at a threshold of 9 mm. The Cox proportional hazards model was used to determine baseline variables associated with the occurrence of stent-graft surface movement. The association between migration and srEL with stent-graft surface movement was tested with the chi-square and the Fisher exact test, respectively.

**Results:**

Stent-graft surface movement was observed in 54 (28.9%) of 187 patients. Multivariate analysis revealed that age ([HR] 1.05; *p* = 0.017), proximal neck diameter ([HR] 5.07; *p* < 0.001), infrarenal aortic neck angulation ([HR] 1.02, *p* = 0.002), and proximal neck length ([HR] 0.62, *p* < 0.001) were significantly associated with the occurrence of stent-graft surface movement. Migration and srEL occurred in 17 (31.5%) and 5 (9.3%) patients, with and 11 (8.3%) and 2 (1.5%) without stent-graft surface movement (*p* < 0.001, *p* = 0.022).

**Conclusions:**

Age, neck diameter, infrarenal neck angulation, and proximal neck length were significantly associated with the occurrence of stent-graft surface movement. Apart from possible use of adjunctive sealing systems, concerned patients may benefit from regular CTA surveillance, enabling timely diagnosis of subtle changes of stent-graft position.

**Key Points:**

*• Stent-graft surface movement, demonstrating subtle, three-dimensional changes in stent-graft position in the proximal anchoring zone, can be derived from CTA examinations.*

*• Age, proximal neck diameter, and infrarenal neck angulation were significantly associated with an increased incidence of stent-graft surface movement. Stent-graft surface movement is significantly more frequent in patients with stent-graft migration and stent-graft-related endoleaks.*

*• Consideration of risk factors for stent-graft surface movement may help to identify patients who might benefit from regular CTA surveillance and timely diagnosis of subtle changes of stent-graft position, enabling re-interventions to prevent migration and srEL.*

**Electronic supplementary material:**

The online version of this article (10.1007/s00330-019-06282-w) contains supplementary material, which is available to authorized users.

## Introduction

The robustness of the overlapping area between the aortic wall and the stent-graft is a determining factor for the long-term durability of endovascular aortic repair (EVAR) [1, 2]. Stent-graft migration has a reported prevalence ranging from 1.1 to 28% [2–5]. It is responsible for the majority of late complications after EVAR, including late stent-graft-related endoleaks, resulting in aneurysm sac enlargement, and even rupture [6, 7]. Different mechanisms, such as the radial forces of self-expandable stent-grafts due to oversizing [8, 9] and the pulsatile forces of blood flow [10–12], were suggested to be associated with continuous changes in stent-graft position and decreasing apposition of the stent-graft surface, consequentially causing migration over time. In addition, disease progression could trigger and accelerate both mechanisms [13, 14]. Stent-graft dynamics over time are complex and three-dimensional [15–17], and not only limited to a one-dimensional, most commonly caudal displacement of the stent-graft, as defined for migration [18]. Longitudinal displacement may occur simultaneously or consecutively with stent-graft surface movement in other directions [16, 19]. Focusing only on caudal migration, stent-graft surface movement in other directions, as a result of subtle aneurysm neck changes, may be overlooked. Diagnosis of these subtle changes on regular computed tomography angiography (CTA) images is difficult, even with centerline reconstructions on a vascular workstation [16, 19]. On the contrary, the calculation of three-dimensional (3D) surface models, derived from CTA images, enables a simplified visualization and assessment of stent-graft surface movement [15, 19], with the potential of a more sensitive determination of changes of the stent-graft position within the infrarenal aortic neck. Therefore, knowledge of the predisposing factors associated with the occurrence of stent-graft surface movement may influence the pre-operative planning in terms of patient selection for EVAR, the possible use of adjunctive sealing systems, and surveillance strategy. Detection of stent-graft surface movement during CTA surveillance, particularly in patients at risk, may allow for planning of timely prophylactic re-interventions to prevent stent-graft migration and consecutive sealing loss.

The primary objective of this study was to evaluate the influence of baseline clinical and morphological parameters on the probability of stent-graft surface movement in patients undergoing EVAR. The second aim was to investigate the association of stent-graft surface movement with migration and stent-graft-related endoleaks (type 1 and 3), representing the leading causes of rupture after EVAR [20, 21].

## Materials and methods

### Study design

This was a retrospective single-center, cross-sectional study of patients with abdominal aortic aneurysm (AAA) who were followed after elective EVAR. The institutional review board (No. 1843/2018) approved the study protocol and waived written, informed consent.

### Study population

Our institutional database was screened for patients who underwent elective endovascular AAA repair between May 2002 and July 2014 and who met the following inclusion criteria: (1) EVAR with bifurcated stent-grafts without intra-procedure implantation of proximal adjunctive sealing devices and (2) available standardized CTA, (a) pre-procedure, within 3 months prior to intervention, (b) pre-discharge within 1 week after intervention, and (c) after a time interval of at least 6 months.

**Fig. 1:**
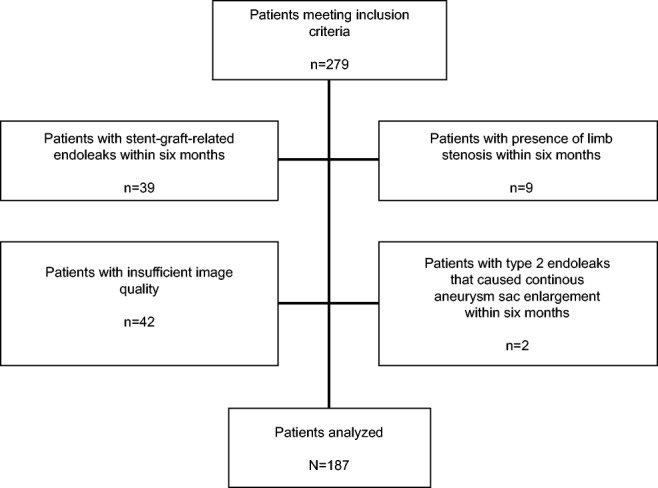
Flow diagram showing patient selection

All patient data were anonymized and de-identified prior to analysis. One hundred eighty-seven patients (14 female) with a mean age of 73.2 ± 8.0 (range, 52–90) years were available for final analysis. Details of patient selection and patient characteristics are given in Fig. 1 and Table 1, respectively. A part of the study population was included in a previously described cohorts [15, 46]. In contrast to our study, analyses referred on the comparison of stent-graft surface movement in patients with and without a type 2 endoleak, and clinical and morphological parameters for the prediction of late stent-graft related endoleaks, respectively.

**Table 1:** Patient characteristics

	*N* (%)
Hypertension	183 (97.9%)
Hyperuricemia	67 (35.8%)
Hyperlipidemia	139 (74.3%)
History of stroke	27 (14.4%)
PAOD (Fontaine stage)	35 (18.7%)
Stage I	2 (1.1%)
Stage IIa	7 (3.7%)
Stage IIb	18 (9.6%)
Stage III	3 (1.6%)
Stage IV	5 (2.7%)
Atrial fibrillation	27 (14.4%)
Cardiac pacemaker	9 (4.8%)
Coronary heart disease	92 (49.2%)
MCI	65 (34.8%)
Diabetes mellitus	32 (17.1%)
IDDM	5 (2.7%)
NIDDM	27 (14.4%)
Renal insufficiency, mild to moderate	58 (30.0%)
Hemodialysis	2 (1.1%)
Smoking	79 (42.2%)
History of cancer	43 (23.0%)

*PAOD* peripheral artery occlusive disease, *MCI* myocardial infarction, *IDDM* insulin-dependent diabetes mellitus, *NIDDM* noninsulin-dependent diabetes mellitus

### CTA examination protocol and stent-graft surface movement

CTAs were performed in the caudo-cranial direction using a 16-slice (Somatom Sensation 16, Siemens Healthineers; *n* = 122) or a dual-source (Somatom Definition Flash; Siemens Healthineers; *n* = 65) scanner. The institutional standard protocol included an arterial phase, ranging from the celiac trunk to the groin solely for pre-interventional imaging, followed by a late phase limited to the extent of the stent-graft in case of postinterventional imaging. Acquisition parameters are given in Table 2.

**Table 2:** Acquisition parameters

	16-slice scanner	Dual-source scanner
Contrast media, ml	Biphasic: 30 and 85	110
Injection rate, ml/s	Biphasic: 6 and 4.5	6
Arterial phase	6 s after threshold of 110 HU*	15 s after threshold of 150 HU*
Venous phase	16 s after arterial phase	18 s after arterial phase
Tube voltage (kV)	120	Ref 120 (Care kV)
Tube current (refmAs, CD4D)	120	120
Detector size/collimation (mm)	16 × 0.75	2 × 64 × 0.6
Rotation time (s)	0.5	0.28
Pitch	≈ 1	≈ 1
Soft kernel	B30	B30
Slice thickness/increment (mm)	1/0.8	1/0.8
FOV	≈ 300	≈ 300
Spatial resolution, *x*/*y* plane	2% MTF 14.7 lp/cm (± 10%)	2% MTF 16.4 lp/cm (± 10%)
Spatial resolution, *z* plane	2% MTF 14.7 lp/cm (± 10%)	2% MTF 18.5 lp/cm (± 10%)

*ml* milliliters, *s* seconds, *HU* Hounsfield units, *kV* kilovolt, *refmAs* reference milliampereseconds, *mm* millimeters, *FOV* field of view, *MTF* modulation transfer function, *lp/cm* line pairs per centimeter

*Region of interest positioned in the aorta at the level of the celiac trunk (bolus tracking technique)

Stent-graft surface movement was assessed as described by Nolz et al [15]. In short, three-dimensional (3D) surface models of the stent-graft, delimited by landmarks using custom-built software, were derived from arterial CTA images. The software calculated a vector for each point of the stent-graft surface, describing the surface movement between the postprocedure and final follow-up CTA, which was given in millimeters. Based on results of previous studies [15, 22], where significantly higher rates of EVAR failure were reported in patients with a stent-graft movement more than 9 mm, this threshold was considered significant.

### Measurements and definitions

The influence of baseline clinical and morphological parameters on the probability of stent-graft surface movement > 9 mm in the proximal anchoring zone was analyzed. As previously reported [15], this zone was defined as the proximal 3 cm of the covered stent-graft.

Baseline clinical parameters included age (at the time of EVAR), sex, and body mass index (BMI). Baseline morphological parameters included anatomical variables measured in the pre-procedural CTA scans, device-dependent variables collected from the pre-interventional CTA and/or the stent-graft procedure, and early postinterventional variables assessed on the pre-discharge CTA. Measurements were performed with the syngo.via imaging software (Siemens Healthineers).

Anatomical variables determined in accordance with the Society for Vascular Surgery standards for EVAR [23] included (1) aneurysm sac diameter, (2) maximum diameter of the proximal anchoring zone, (3) iliac sealing diameters, and (4) proximal and distal neck lengths. The supra- and infrarenal aortic neck angulations were measured based on the 2D methodology described by van Keulen et al [24], and adapted for measuring along a semi-automatically drawn center lumen line (CLL). Assessment of the psoas muscle area (PMA) was performed as described by Indrakusuma et al [25]. The area of both psoas muscles was added and corrected for patient height using the formula (left PMA + right PMA)/(height^2^) [26]. This variable was determined as height-corrected PMA.

The presence of a thrombus with > 2 mm thickness at the circumference of the proximal anchoring zone was evaluated and classified as follows: (1) no thrombus, (2) thrombus < 25%, (3) thrombus 25–50%, or (4) thrombus ≥ 50%. The presence of calcifications was categorized according to the same classification. On the basis of gathered anatomical variables, compliance requirements with the manufacturer’s instructions for use (IFU) were analyzed, and rated as within or outside the IFU.

The followed devices were implanted: Talent (Medtronic; *n* = 36, 19.3%), Excluder (W.L. Gore & Associates; *n* = 74, 39.6%), Zenith (Cook Medical; *n* = 20, 10.7%), Endurant (Medtronic; *n* = 46, 24.6%), Anaconda (Vascutek; *n* = 4, 2.1%), Aorfix (Lombard Medical Technologies; *n* = 1, 0.5%), Powerlink (Endologix, Inc.; *n* = 1, 0.5%), and Treovance (Bolton Medical; *n* = 5, 2.7%). Device-dependent variables included (1) proximal oversizing factor, (2) distal oversizing factors, (3) proximal fixation level (suprarenal/infrarenal), (4) implantation side of the modular limb (right/left), and (5) presence of any active fixation mechanism (e.g., hooks, anchoring pins, or dull barbs).

Early postinterventional variables included (1) the presence of a type 2 endoleak (T2EL) and (2) the renal artery to stent-graft distance (RSD). RSD was measured along a semi-automatically drawn CLL, as described by Bastos Goncalves et al [27]. RSD was defined as the distance between the lowest renal artery to the lowest stent-graft fabric marker, representing the level of circumferential stent-graft covering of the aortic wall. RSD changes between both examinations indicate stent-graft migration, which was defined as an RSD increase of > 0.5 cm [28]. Routinely performed CTA follow-up examinations were retrospectively screened for the presence of a stent-graft-related endoleak, type 1 or type 3, as described by Chaikof et al [18].

### Statistical analysis

Normally distributed, continuous data were presented as the mean ± standard deviation. Potential differences between groups were compared using the *t* test. Non-normally distributed data were described by medians and interquartile ranges (IQRs). Possible differences between groups were tested with the Wilcoxon-Mann-Whitney *U* test. Dichotomous variables were described in absolute numbers and percentages, and possible differences between groups were tested by the chi-square test or the Fisher exact test, as appropriate. The univariate Cox proportional hazards model, with calculation of hazard ratios with 95% confidence intervals, was used to investigate the influence of baseline clinical and morphological parameters on the probability of stent-graft surface movement-free survival. A multivariate Cox proportional hazards model was conducted by using a backward selection of parameters, with a limit of *p* < 0.1 required to enter and to stay in the model. The proportional hazards assumption was tested for each variable individually using the time-dependent covariate method [29]. No relevant violations of the assumption were found (see supplementary material). The effect of adding interaction terms was assessed using the partial likelihood ratio test. No statistically significant interactions were determinable. Additionally, receiver operating characteristics (ROC) analysis was performed in variables, significantly associated with stent-graft surface movement in the multivariate analysis. Cut-off values were defined with the Youden *J* statistics. The Kaplan-Meier life table method was used to determine freedom from stent-graft surface movement, migration, and stent-graft-related endoleaks. For these survival analyses, all observations were censored at the time of the patient’s last CTA. All tests were two-sided; significance was assumed at *p* < 0.05. All statistical analyses were performed using SPSS for Windows (version 24.0; IBM Corporation).

## Results

**Fig. 2:**
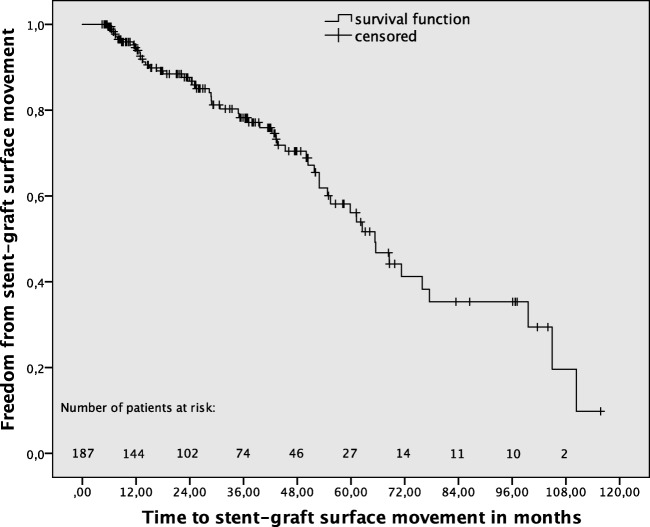
Kaplan-Meier survival curve demonstrating freedom from stent-graft surface movement

**Fig. 3:**
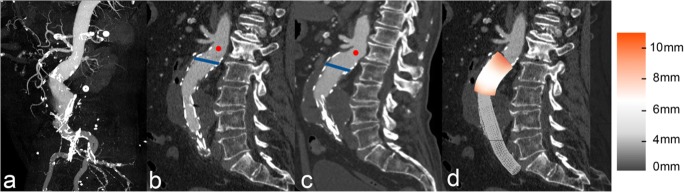
**a**–**d** 92-year-old male patient with severe angulation and large diameter (28 mm) of the infrarenal neck on pre-procedural CTA-maximum intensity-projection images (**a**). Comparing sagittal pre-discharge (**b**) and 3-year follow-up (**c**) CTA images, widening of the proximal aortic neck diameter and significant migration (RSD 13 mm vs 25 mm) were observed. The red dot (**b**, **c**) marks the lowest renal artery, while the blue line represents fabric markers of the stent-graft coverage. Significant stent-graft surface movement (> 9 mm) occurred at the proximal landing zone, visualized by 3D matched surface models of postprocedural and final follow-up CTA (**d**)

**Fig. 4:**
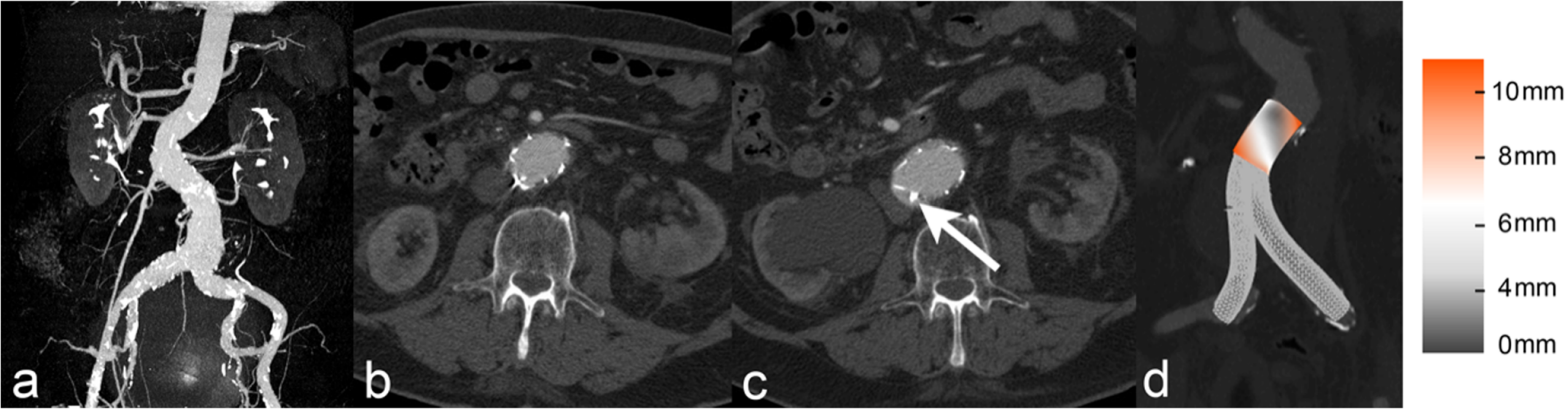
**a**–**d** 83-year-old male patient with evident angulation and wide diameter of the infrarenal neck on pre-procedural CTA-maximum intensity-projection images (**a**). In comparison to the pre-discharge CTA (**b**), there is an obvious lateralization of the superior mesenteric artery and the abdominal aorta with consecutive compression of the inferior vena cava after 4 years of follow-up (**c**). Further, the aortic neck diameter increased from 29 to 36 mm with consecutive sealing loss at the dorsal circumference of the proximal anchoring zone and occurrence of a type 1a endoleak (arrow). Significant stent-graft surface movement (> 9 mm) at the proximal anchoring zone is visualized on 3D matched surface models of postprocedural and final follow-up CT (**d**)

**Fig. 5:**
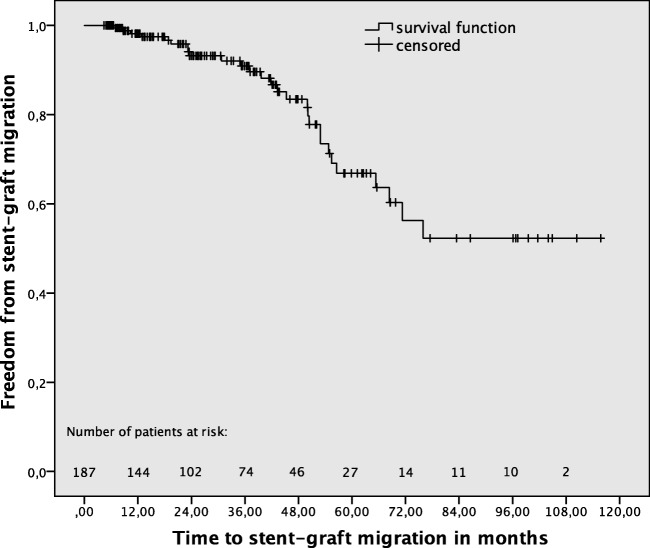
Kaplan-Meier survival curve demonstrating freedom from stent-graft migration

**Fig. 6:**
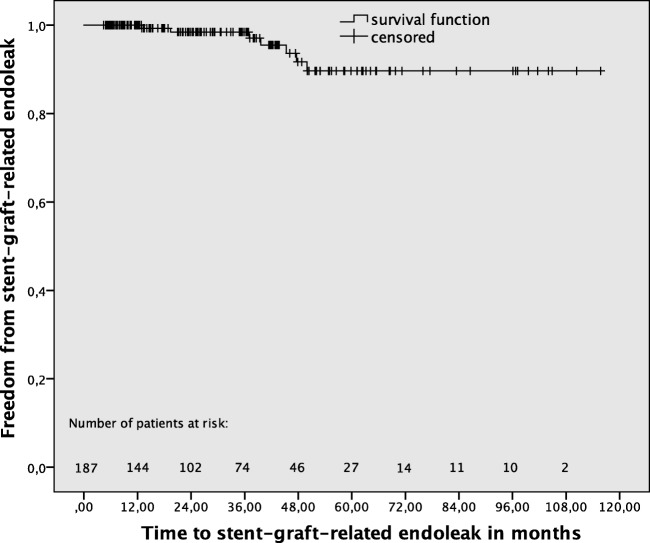
Kaplan-Meier survival curve demonstrating freedom from stent-graft-related endoleak

Stent-graft surface movement more than 9 mm was detected in 54 (28.9%) of 187 patients during a mean MSCTA follow-up of 33.5 ± 25.4 (median and IQR) months. Overall, cumulative freedom from stent-graft surface movement rates after 1, 3, and 5 years were 94.6%, 78.8%, and 55.3%, respectively (Fig. 2). Stent-graft migration occurred significantly (*p* < 0.001) more frequently in patients with (*n* = 17, 31.5%), compared to those without (*n* = 11, 8.3%), stent-graft surface movement (Fig. 3a–d). Late srEL occurred significantly (*p* = 0.022) more frequently in patients with (*n* = 5, 9.3%), compared to those without (*n* = 2, 1.5%), stent-graft surface movement (Fig. 4a–d). Cumulative freedom from stent-graft migration and stent-graft-related endoleak rates after 1, 3, and 5 years were 98.1%, 90.9%, and 66.8%, as well as 100%, 98.5%, and 89.6%, respectively (Figs. 5 and 6).

There were four (1.7%) type 1a endoleaks caused by stent-graft migration that resulted in rupture in two patients, 45 and 42 months after treatment. Successful redo-EVAR was performed by implantation of an aorto-mono-iliac device implanted in one patient, and a bifurcated endoprosthesis in the second patient. The two other type 1a endoleaks were treated by implantation of proximal extensions (Endurant, Medtronic). Furthermore, two patients with a left-sided modular limb suffered from type 1b endoleaks (one left, one right), which were treated by extension of the iliac limb into the external iliac artery after coil occlusion of the internal iliac artery. One type 3 endoleak, caused by disconnection of the left modular limb, was over-stented. Baseline clinical and morphological parameters of the entire cohort, separated for patients with and without stent-graft surface movement, are given in Table 3. Factors with a significance level < 0.1 in the univariate analysis associated with stent-graft surface movement are given in Table 4. Multivariate analysis revealed that age ([HR] 1.05, 95% CI 1.01–1.08; *p* = 0.017), proximal neck diameter ([HR] 5.07, 95% CI 1.94–13.23; *p* < 0.001), and infrarenal aortic neck angulation ([HR] 1.02, 95% CI 1.01–1.03; *p* = 0.002) were significantly associated with a higher incidence of stent-graft surface movement. Proximal neck length ([HR] 0.62, 95% CI 0.49–0.80; *p* < 0.001) proved to be significantly associated with a decreased incidence of stent-graft surface movement in the multivariate analysis (Table 4).

**Table 3:** Baseline clinical and morphological parameters

	Overall (*n* = 187)	Stent-graft surface movement (*n* = 54)	No stent-graft surface movement (*n* = 133)	*p*
Baseline clinical variables				
Sex, female	14 (7.5%)	5 (9.3%)	9 (6.8%)	0.550
Age, years	73.22 ± 7.97	74.19 ± 7.72	72.83 ± 8.06	0.288
Body mass index, kg/m^2^	28.18 ± 4.87	28.62 ± 5.57	28.00 ± 4.56	0.427
Baseline anatomical variables				
Aneurysm sac diameter, cm	5.99 ± 0.90	6.07 ± 0.97	5.96 ± 0.87	0.451
Proximal neck length, cm	3.08 ± 1.28	2.63 ± 1.15	3.26 ± 1.30	0.002*
Proximal neck diameter, cm	2.42 ± 0.31	2.50 ± 0.29	2.39 ± 0.31	0.029*
Right iliac sealing diameter, cm	1.25 ± 0.22	1.24 ± 0.22	1.25 ± 0.22	0.818
Right iliac neck length, cm	1.72 ± 0.56	1.72 ± 0.52	1.72 ± 0.58	0.961
Left iliac sealing diameter, cm	1.21 ± 0.23	1.24 ± 0.26	1.20 ± 0.22	0.261
Left iliac neck length, cm	1.85 ± 0.55	1.85 ± 0.61	1.85 ± 0.52	0.997
Suprarenal aortic neck angulation, °	31.53 ± 21.57	29.85 ± 21.18	32.22 ± 21.76	0.498
Infrarenal aortic neck angulation, °	34.67 ± 23.52	36.93 ± 26.11	33.76 ± 22.24	0.406
Height-corrected PMA, cm^2^/m^2^	5.52 ± 1.47	5.52 ± 1.62	5.51 ± 1.41	0.968
No neck thrombus	80 (42.8%)	15 (27.8%)	65 (48.9%)	0.008*
< 25%	32 (17.1%)	12 (22.2%)	20 (15.0%)	
25–50%	44 (23.5%)	18 (33.3%)	26 (19.5%)	
≥ 50%	31 (16.6%)	9 (16.7%)	22 (16.5%)	
No neck calcification	163 (87.2%)	47 (87.0%)	116 (87.2%)	0.973
< 25%	19 (10.2%)	6 (11.1%)	13 (9.8%)	
25–50%	4 (2.1%)	1 (1.9%)	3 (2.3%)	
≥ 50%	1 (0.5%)	0	1 (0.8%)	
IFU, outside	39 (20.9%)	16 (29.6%)	23 (17.3%)	0.060
Device-dependent variables				
Proximal oversizing, %	16.93 ± 6.94	16.46 ± 6.50	17.12 ± 7.12	0.556
Right distal oversizing, %	17.00 ± 7.82	18.29 ± 9.41	16.47 ± 7.04	0.150
Left distal oversizing, %	17.94 ± 9.64	17.04 ± 8.20	18.30 ± 10.18	0.421
Fixation level, suprarenal	107 (57.2%)	38 (70.4%)	69 (51.9%)	0.021*
Fixation level, infrarenal	80 (42.8%)	16 (29.6%)	64 (56.6%)	
Active fixation mechanism	150 (80.2%)	37 (68.5%)	113 (85.0%)	0.011*
No active fixation mechanism	37 (19.8%)	17 (31.5%)	20 (15.0%)	
Implantation side of modular limb, left	146 (78.1%)	40 (74.1%)	106 (79.7%)	0.399
Early postinterventional variables				
Type 2 endoleak	94 (50.3%)	21 (38.9%)	73 (54.9%)	0.047*
Lumbar	53 (28.3%)	10 (18.5%)	43 (32.3%)	
Lumbar + IMA	20 (10.7%)	4 (7.4%)	16 (12.0%)	
IMA	20 (10.7%)	7 (13.0%)	13 (9.8%)	
Accessory renal artery	1 (0.5%)	0	1 (0.8%)	
RSD, mm	0 (IQR 0–0.40)	0 (IQR 0–0.43)	0 (IQR 0–0.40)	0.575

Comparisons between groups were performed with the *t* test, the Wilcoxon-Mann-Whitney *U* test, the chi-square test, or the Fisher exact test, as appropriate

*PMA* psoas muscle area, *IMA* internal mesenteric artery, *RSD* renal atery to stent-graft distance, *p* patients with versus without stent-graft surface movement

*Indicates a significant difference

**Table 4:** Results of Cox regression analysis to identify baseline factors associated with stent-graft surface movement

*N* = 187	Univariate	Wald test	Multivariate	Wald test
Co-variable	Hazard ratio	*p*	Hazard ratio	*p*
Age, years	1.04 (1.01–1.08)	0.023*	1.05 (1.01–1.08)	0.017*
Aneurysm sac diameter, cm	1.32 (0.96–1.80)	0.085		
Proximal neck length, cm	0.68 (0.53–0.87)	0.002*	0.62 (0.49–0.80)	< 0.001*
Proximal neck diameter, cm	4.04 (1.66–9.86)	0.002*	5.07 (1.94–13.23)	0.001*
Infrarenal aortic neck angulation, °	1.01 (1.00–1.02)	0.064	1.02 (1.01–1.03)	0.002*
Proximal fixation level suprarenal	2.68 (1.46–4.90)	0.001*		
Proximal oversizing, %	0.96 (0.93–1.00)	0.065		
IFU, outside	1.96 (1.08–3.54)	0.027*		
Left iliac diameter, cm	2.82 (0.95–8.38)	0.062		

*IFU* instructions for use

*Indicates a significant difference

For identification of patients with stent-graft surface movement, the optimal cut-off points for age, neck diameter, neck length, and infrarenal neck angulation were 72.3 years, 2.65 cm, 4.15 cm, and 39.5° with areas under the curve of 0.538 (95% CI 0.449–0.628; *p* = 0.410), 0.607 (95% CI 0.519–0.695; *p* = 0.022), 0.633 (95% CI 0.546–0.719; *p* = 0.004), and 0.530 (0.435–0.625; *p* = 0.526), respectively.

## Discussion

The present study analyzed the influence of baseline clinical and morphological parameters on the occurrence of stent-graft surface movement after endovascular aneurysm repair. A multivariate Cox model revealed a relation between stent-graft surface movement and patient age, the proximal aneurysm neck diameter as well as the infrarenal aortic neck angulation, whereas proximal neck length proved to be significantly associated with a decreased incidence of stent-graft surface movement.

Furthermore, stent-graft migration and srEL occurred significantly more frequently in patients with stent-graft surface movement.

EVAR should not be considered to be a single-step procedure, based on preoperative anatomical features alone. During pre-procedure planning, one should be aware of the ongoing process of conformational geometrical changes of the stent-graft and the diseased aorta [30, 31]. In this context, an intact proximal sealing zone is of crucial importance for stent-graft stability and integrity, preventing distal migration, stent-graft-related endoleaks, and, ultimately, aortic rupture. However, detection of these subtle changes on regular CTA images is difficult, even with centerline reconstructions on a vascular workstation [16, 19]. A recent study by Schuurmann et al [19], using a semiautomatic software, observed significant changes in proximal stent-graft dimension and apposition on regular CTA scans prior to failure, when patients with and without later stent-graft migration were compared. This observation was reflected in our results, where subtle changes of the stent-graft surface could be detected in 28.9% of patients using a custom-made semi-automatic software. Consistently, migration and srEL were significantly more frequent in patients with stent-graft surface movement.

The incidence of stent-graft migration in the present study was 15.0%, which is in line with percentages (1.1–28%) previously reported [2–5]. Migration bears the risk of sealing loss followed by late stent-graft-related endoleaks, which were observed in 3.7% of our patients. This incidence is lower compared with the percentages (3.9–10.6%) previously reported [32–34], which may be explained by the exclusion of patients with early srEL and re-intervention within 6 months.

Different anatomical aspects of proximal neck morphology were found to be associated with the incidence of migration. In this context, the aortic neck diameter was found to be a risk factor for stent-graft migration [3, 4, 35], which is in accordance with our results where an increasing neck diameter was related to stent-graft surface movement in the multivariate analysis. Shorter neck length is a well-known risk factor for migration [36, 37], which is in line with our results, demonstrating the protective effect of longer neck lengths in terms of stent-graft surface movement. Another feature of hostile neck anatomy is angulation, which decreases the necessary pull-down force causally responsible for dislodgement of the stent-graft [38]. Consequently, neck angulation was found to be a risk factor for early migration [32] and type 1 endoleaks [39]. This mechanism is supported by our results, where neck angulation was found to be significantly associated with stent-graft surface movement in the multivariate analysis. Displacement forces and stent-graft stability are also related to the iliac fixation [14, 37]. Through distal fixation, different authors have identified the docking area of the modular stent graft limb as a vulnerable region, which seems to be exposed to the highest mechanical stress [31] and surface movement [15]. Taking into account that the modular limb was predominantly left-sided (78%) in our cohort, the left iliac sealing diameter was significantly associated with an increased stent-graft surface movement in the univariate, but not in the multivariate, analysis.

Another mechanism with a negative influence on wall compliance and aneurysm morphology is atherosclerotic disease progression [40], which particularly affects elderly patients [41]. Several authors reported an association between early stent-graft complications, including migration [42, 43], and increasing age. These findings were supported by our data, where increasing age was significantly associated with stent-graft surface movement in the multivariate analysis.

Different approaches were made to streamline the surveillance protocol proposed by the Society of Vascular Surgery [18], designed to reduce radiation exposure and the total costs of EVAR. As outlined by Hoel and Schanzer [44, 45], there should be a balance between adequate disease detection and unnecessary use of resources in postoperative surveillance. Knowledge of the predisposing factors associated with the occurrence of stent-graft surface movement may help to identify patients who benefit from regular CTA surveillance. Detection of subtle changes of the stent-graft position in these patients may allow for planning of timely prophylactic re-interventions to prevent stent-graft migration and consecutive sealing loss [19].

### Limitations

Our study has several limitations. First, data were evaluated in a retrospective manner and we had to exclude 50 patients with early re-interventions for stent-graft failure, rendering the assessment of stent-graft surface movement impossible. Since we have no information on possible late complications of these patients, we cannot completely rule out a possible selection bias. Second, inclusion of second-generation devices and rudimentary planning in the early period of our study may have negatively affected the outcome in certain patients. Third, comparing the pre-discharge and the last CTA follow-up resulted in a heterogeneous time interval for measurements. As a consequence, the study design does not allow a conclusion about the time point for the occurrence of stent-graft surface movement and migration, nor on its chronological sequence. Fourth, due to the relatively small sample size and heterogeneity by means of implanted devices, we are not able to draw a reasonable statement on the performance of different devices.

## Conclusion

Older patients, those with larger aneurysm neck diameters, and those with increased infrarenal aortic neck angulation experienced higher rates of stent-graft surface movement. Consideration of these risk factors may help to identify patients who benefit from possible use of adjunctive, prophylactic sealing systems and a regular CTA surveillance or even open repair as a primary intervention. Detection of stent-graft surface movement during CTA surveillance may allow for planning of timely prophylactic re-interventions to prevent stent-graft migration and stent-graft-related endoleaks.

## Electronic supplementary material

ESM 1 (DOCX 18 kb)

## Abbreviations

AAA: Abdominal aortic aneurysm
BMI: Body mass index
CTA: Computed tomography angiography
EVAR: Endovascular aneurysm repair
IFU: Instructions for use
PMA: Psoas muscle area
RSD: Renal artery to stent-graft distance
srEL: Stent-graft-related endoleak
T2EL: Type 2 endoleak
