# Supplementary material for: Stent-graft surface movement after endovascular aneurysm repair: baseline parameters for prediction, and association with migration and stent-graft-related endoleaks
Source: Eur Radiol. 2019 Jun 27;29(12):6385–95. doi: 10.1007/s00330-019-06282-w (PMC6828830; doi:10.1007/s00330-019-06282-w)
Supplement: Supplementary file 1 — (DOCX 18 kb) [file 330_2019_6282_MOESM1_ESM.docx]

Test of the proportional hazards assumption

| Co-variable | Hazard Ratio | Wald test, p = |
| --- | --- | --- |
| T_COV x age | 1.00 (0.99 – 1.00) | 0.275 |
| T_COV x Aneurysm sac diameter | 0.99 (0.98 – 1.01) | 0.587 |
| T_COV x Proximal neck length | 1.00 (0.99 – 1.01) | 0.977 |
| T_COV x Proximal neck diameter | 1.03 (0.99 – 1.07) | 0.184 |
| T_COV x Infra-renal aortic neck angulation | 1.00 (0.99 – 1.00) | 0.211 |
| T_COV x Proximal fixation level suprarenal | 1.01 (0.99 – 1.04) | 0.250 |
| T_COV x Proximal oversizing | 0.99 (0.99 – 1.00) | 0.493 |
| T_COV x IFU, outside | 0.98 (0.96 – 1.01) | 0.123 |
| T_COV x Left iliac diameter | 1.00 (0.95 – 1.05) | 0.996 |

IFU, instructions for use; * indicates a significant difference
